# Improving the mechanical behavior of reduced graphene oxide/hydroxyapatite nanocomposites using gas injection into powders synthesis autoclave

**DOI:** 10.1038/s41598-020-64928-y

**Published:** 2020-05-22

**Authors:** Hassan Nosrati, Rasoul Sarraf-Mamoory, Dang Quang Svend Le, Reza Zolfaghari Emameh, Maria Canillas Perez, Cody Eric Bünger

**Affiliations:** 1 Department of Materials Engineering, Tarbiat Modares University, Tehran, Iran; 2 Department of Clinical Medicine, Aarhus University, Aarhus, Denmark; 3 Department of Energy and Environmental Biotechnology, National Institute of Genetic Engineering and Biotechnology (NIGEB), 14965/161 Tehran, Iran; 4 Instituto de Cerámica y Vidrio, CSIC, Madrid, Spain

**Keywords:** Biomaterials, Nanoscale materials

## Abstract

In this study, we show the synthesis of reduced graphene oxide/hydroxyapatite (rGO/HA) composites using a hydrothermal autoclave with argon-15% hydrogen gas injection. This both increases the hydrothermal pressure and uses hydrogen as a reductive agent in the process. The synthesized powders were then consolidated with spark plasma sintering method. The analysis of the consolidated samples included Vickers Indentation technique and cell viability. The results showed that injected gases in the autoclave produced powders with a higher crystallinity compared to synthesis without the gases. Also, hydrogen gas led to increased reduction of GO. The microscopic analysis confirmed existing graphene sheets with folding and wrinkling in the powders and indicated that various preferential directions played a role in the growth of hydroxyapatite crystals. The results showed that in general, graphene sheets increased the mechanical properties of HA. In the samples synthesized with injected gases, this increase was more significant. Interface analysis results indicate that reduced graphene oxide (rGO)/HA interface is likely coherent. These nanocomposites were biocompatible and showed some hydrophobicity compared to pure HA.

## Introduction

Hydroxyapatite (HA), a member of the calcium phosphate family, is one of the most valuable biomaterials used in orthopedics^1^. The unique properties of HA include excellent bioactivity, osteoconductivity, and biocompatibility. The chemical formula of this ceramic is Ca_10_(PO_4_)_6_(OH)_2_, in which the ratio of calcium to phosphate is 1.67, and the crystalline structure of HA is similar to the inorganic part of the human skeletal system. HA has a variety of uses other than orthopedic applications^2–5^. In recent years, additional researches have been done on calcium phosphates, which indicate the specific value of these ceramic structures^6–9^. Despite the excellent biomaterial properties the poor mechanical behavior of HA has limited its application scope. These mechanical properties include brittleness, fracture toughness, and wear resistance, which are important in applications as implants^10–12^.

With the advent of nanotechnology and advanced synthesis methods, the mechanical properties of HA have improved somewhat by decreasing their particles size and morphologies such as nanorods and nanotubes^13–15^. But this improvement is not enough for applications as implants, and the mechanical properties of HA still need to be increased by adding a reinforcement phase^16,17^. Of all the materials used, carbon nanomaterials such as nanotubes^18,19^ and graphene^20^ have recently received much attention and among the carbon materials, graphene and its derivatives have gained more research because of their unique properties. Graphene has a honeycomb-like structure. Graphene sheets are as thick as a carbon atom. The atoms in the graphene structure are combined with the sp^2^ hybrid^21–23^. The mechanical properties of graphene are very interesting, which include the elastic modulus of 1 TPa and the fracture strength of 130 GPa^24,25^. The high reinforcing properties of graphene are due to the two-dimensional structure with high specific surface area of 2630 m^2^g^−1^ ^26^. Graphene, having good biological properties, has been extensively studied in biological applications, including drug delivery^27^, biosensing^28^, tissue engineering^29^, and bioimaging^30^.

Several researches have been published on the use of graphene to improve the mechanical and biological properties of HA and the results of these studies have shown that graphene has greatly enhanced the properties of HA^31–34^. Graphene-HA composites are fabricated in various methods. Some of these methods include hydrothermal process^32,35^, electrochemical deposition technique^36,37^, sonochemical treatments^38^, biomineralization^39^, and self-assembly^40^. Some methods are more suitable for coating. In some methods, such as hydrothermal, the hybrid powders are first synthesized and subsequently consolidated by spark plasma sintering (SPS)^41–43^ or hot pressing method^33,44^. Among these methods, the hydrothermal process is very convenient for easier control and the ability to synthesize hybrid powders by in situ method^45,46^. When other solvents are used beside water, this process is called solvothermal method^47^. Graphene oxide is mainly used in this method. Due to the presence of functional agents on the surface of graphene oxide, nucleation and growth of HA crystals are easily accomplished. In the hydrothermal method, high temperature and process time cause the graphene oxide to reduce. This also causes the HA particles to form nanorods on the graphene oxide surface^48,49^. With the reduction of graphene oxide, its mechanical properties improve. Different methods are used to increase the reduction rate including adding chemicals such as ethylene glycol^33^, and injecting hydrogen or nitrogen gas into a hydrothermal autoclave^50–53^. Increasing hydrothermal pressure increases the crystallinity of HA^54^ and increases the rate of graphene oxide reduction^45^.

In this study, a hydrothermal method was used to synthesize reduced graphene oxide- HA nanocomposite powders under the Ar-15% H_2_ mixed gases injection condition. Pure HA was previously synthesized under this condition, and increased pressure by injecting this mixture of gases increased the mechanical properties of HA^54^. The presence of hydrogen gas in this mixture is now exploited to increase the rate of graphene oxide reduction. After the powders were synthesized and characterized (X-ray diffraction, field emission scanning electron microscopy, Fourier transform infrared spectroscopy, Raman spectroscopy, and high-resolution transmission electron microscopy), spark plasma sintering (SPS) was performed and the samples were subjected to mechanical and biological assays. It is expected that the combination of the above methods will make the final nano-structured composite suitable for orthopedic applications.

## Experimental

The primary chemicals used in this study, along with their specifications, are presented in Table 1. The initial solution (S1) was first prepared (diethylene glycol + dimethyl formamide + deionized water with a volume ratio of 10: 10: 80) and the following steps were performed in order. The solution containing Ca^+2^ (4.7 grams of calcium nitrate tetrahydrate in 120 mL of S1) was added dropwise to a 20 mL stirred suspension of GO (HA/1.5% rGO) with stirring continued for 1 h. The solution containing phosphate ions (1.56 grams of diammonium hydrogenphosphate in 80 mL of S1) was dropwise added to the solution. The pH of the solutions was adjusted to >10 with ammonium solution. The resulting solution was poured into the Teflon (PTFE) vessel and transferred to the autoclave. The hydrothermal process was carried out for 8 h at 180 °C by injection of nitrogen gas at 0, 5, and 10 bar (The volume of the PTFE container was 340 mL). The powders were dried at oven for 12 h at 60 °C. The resulting powders were sintered after drying and ball milling (250 rpm, 12 h)^46,48,49^.

**Table 1:** The primary chemical used in the powders synthesis phase.

Chemical	Specification
dimethyl formamide (DMF)	Sigma Aldrich (> 99.8%), (CH_3_)_2_NC(O)H
graphene oxide (GO)	Abalonyx (25 g/L DMF), CO_x_H_y_
diethylene glycol (DEG)	Sigma Aldrich (99%), (HOCH_2_CH_2_)_2_O
calcium nitrate tetrahydrate	Merck (> 99%), Ca(NO_3_)_2_.4H_2_O
diammonium hydrogenphosphate	Merck (> 99%), (NH_4_)_2_HPO_4_
ammonium solution	Merck (25%), NH_4_OH

**Figure 1:**
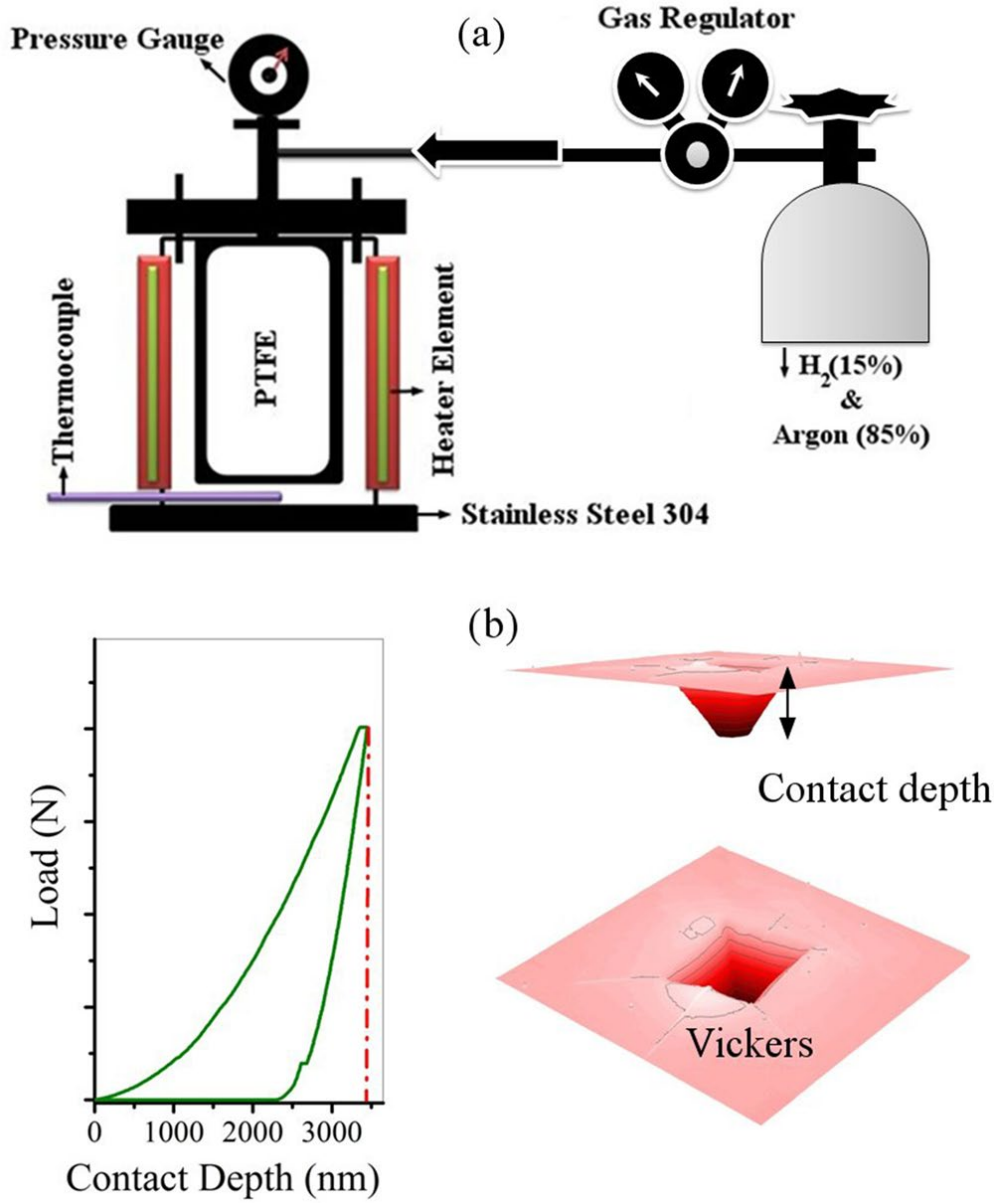
(**a**) hydrothermal system used in this research, (**b**) load-displacement, and indentation affected zone.

Figure 1 shows the hydrothermal system used in this research, load-displacement, and indentation affected zone. Descriptions of this system (Fig. 1a) have already been published^54^.

### Evaluation of the consolidated samples

The powders were consolidated via SPS, as previously reported^54^, at a temperature of 950 °C. Exactly similar to the previous report, the biocompatibility assays were performed^54^.

To calculate the relative density of sintered samples the Archimedes method was used. In this method, wet weight, dry weight and immersed weight with a precision of 0.0001 were measured and the Archimedes ratio was used for relative density calculations (ASTM C373-88)^54^.

### Vickers indentation

Instrumented microindentation experiments (Grindosonic tester with a Vickers tip) were conducted on the polished surfaces of samples at a maximum load of 2 N and ramp dwell time of 10 s. Nine tests were performed at different locations of each sample. Elastic modulus and hardness were calculated from the load-displacement curves (Fig. 1b) using Oliver-Pharr method^55^. The modified Antis method was used to evaluate the fracture toughness (K1C) of the samples (Eq. 1)^56^:

1 $$K1C=\lambda .{({W}_{t}/{W}_{e})}^{0.5}.(P/{C}^{1.5})$$

W_t_ is the area below the load-displacement curve and W_e_ the area below the unloading curve which corresponds to the elastic deformation. The energy W_t_ is the total of elastic and plastic deformation (W_e_ and W_p_ respectively). λ is a dimensionless constant is close to 0.0498 for Vickers tip. C is the average crack length and P is the applied force. The use of experimental parameters is the major advantage of this method; it is easy to calculate when using instrumented indentation.

### Characterization techniques

Different evaluation methods have been used in this research, some of which are described below. The other instruments used to characterize the samples include inductively coupled plasma (ICP) (DV7300, Optima Co.), X-ray photoelectron spectroscopy (XPS, Thermo ESCALAB 250XI), FESEM (SIGMA VP-500, ZEISS), and TEM (CM120, Philips). ImageJ 1.52d and Diamond 3.2 softwares were used in this study.

#### X-ray diffraction

X-ray diffraction (XRD, X′ Pert Pro, Panalytical Co.) was used to determine the phase constituents of the samples, contained a detector Cu Kα radiation (40 kV, 40 mA, λ = 1.5406 Å) and 2 theta from 10° up to 80° in steps of 0.02°. Equation 2 was used to estimate the crystallinity of HA (Xc)^57^.

2 $${X}_{c}=1-\frac{{{\rm{\nu }}}_{(112/300)}}{{{\rm{I}}}_{300}}$$

Where υ_(112/300)_ and I_300_ are the intensity of the hollow between diffraction peaks of HA in the planes (300) and (112) and the intensity of the peak of HA in the plane (300), respectively. Equation 3 was used to calculate the crystallite size (Williamson-Hall method)^58^.

3 $$\beta .Cos\,\theta =\frac{(0.9{\rm{\lambda }})}{{\rm{d}}}+4\varepsilon .Sin\,\theta $$

In this equation, d, θ, and λ are grain size, Bragg diffraction angle, and wave length of used X-ray (Cu), respectively. β and ε are full width at half height (FWHM), and crystalline lattice strain, respectively.

#### Field emission scanning electron microscopy

Field emission scanning electron microscope (FESEM, Hitachi S4700 equipped with energy dispersive X-ray spectroscopy) and a portable scanning electron microscope (SEM, TM-1000) were used to observe the morphology of samples (mounted in an adhesive carbon film and Au coated by sputtering for its observation).

#### Fourier transform infrared spectroscopy

Fourier transform infrared spectroscopy (FTIR, VERTEX 70, Bruker Corp.) was used to identify the functional groups of the samples (resolution of 4 cm^−1^, spectral region from 400 to 4000 cm^−1^ using 2 cm^−1^ steps, scan number of 8). The samples were prepared and mixed with potassium bromide (1 mg powdered samples and 300 mg KBr). The mixture was pressed into discs by applying 200 MPa pressures (1 mm thickness). The spectra were collected at room conditions (60% relative humidity, 25 °C)^48,49^.

#### Raman spectroscopy

Raman spectroscopy (Renishaw inVia spectrometer) was used in the range of 300–3500 cm^−1^, recording 5 times for 10 seconds of each accumulation, with a wavelength of 532 nm, green laser line in a backscattering configuration using a microscope (100× objective, 100% power, an acquisition time of 10 s), which had been excited from an argon ion laser. The samples were subjected on Al foil in order to remove the fluorescence background^48,49^.

#### High-resolution transmission electron microscopy

High-resolution transmission electron microscopy (HRTEM, TALOS F200A with a twin lens system, X-FEG electron source, Ceta 16 M camera and a super-X EDS detector) was used to observe atomic structure of the samples and spatially resolved elemental analysis, with a spatial resolution higher than 2 nm (using TALOS microscope in STEM mode, exposure times of 5 minutes were used to create elemental distribution maps). High angle annular dark field detector (HAADF) was used to obtain STEM images (RG overlays of the STEM EDX elemental maps were made using the FIJI). To study the atomic structure, fast fourier transform (FFT) and inverse fast fourier transform (IFFT) analysis were used^48^.

## Results and discussion

**Figure 2:**
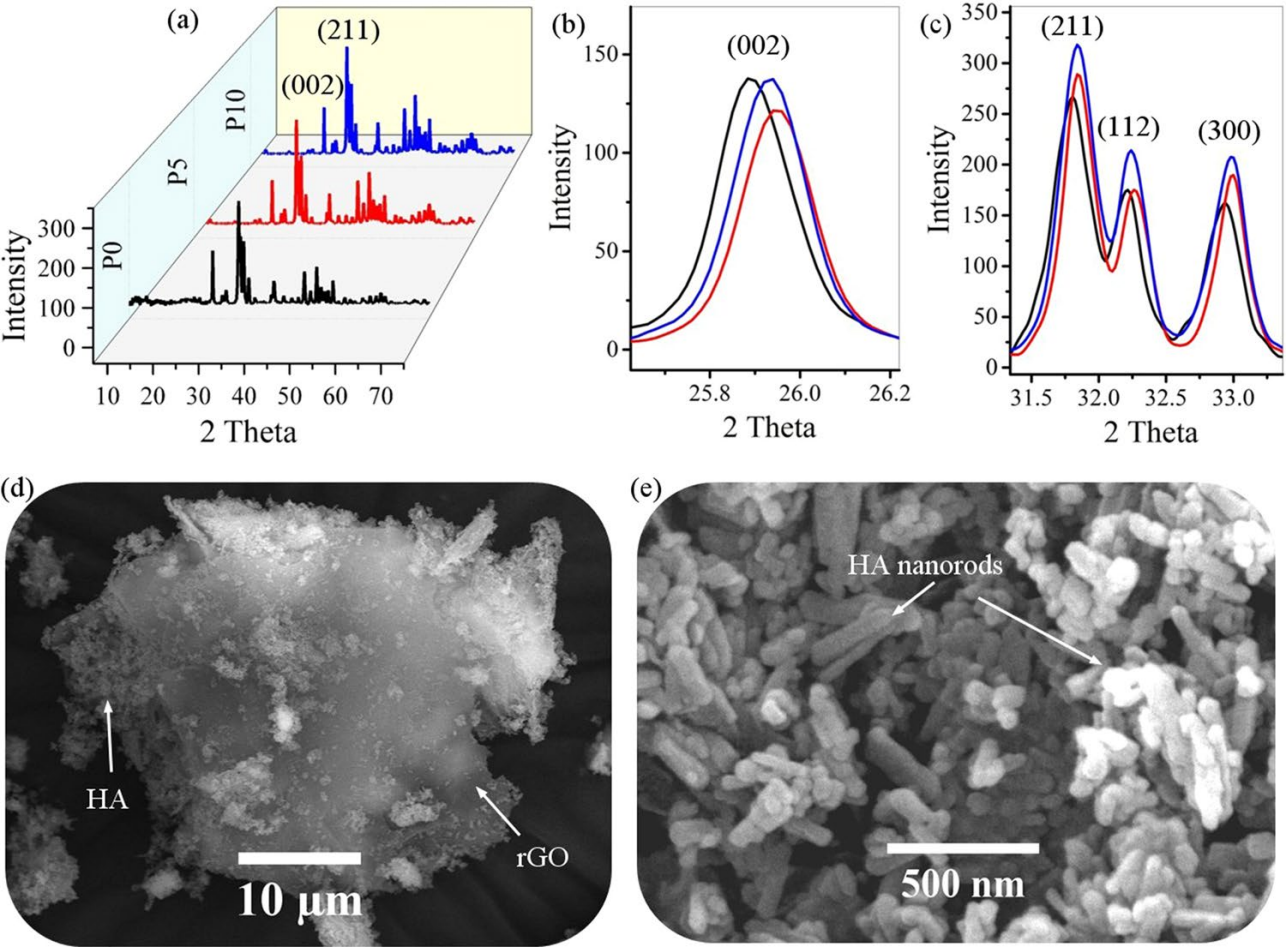
(**a–c**) XRD patterns and (**d,e**) FESEM images of rGO-HA powders (P10).

Figure 2 shows the XRD patterns and FESEM images of rGO-HA (P10) powders. According to the XRD pattern of the synthesized powders (Fig. 2a), full conformity is achieved between the peaks obtained and the reference standard of pure HA (JCPDS 09-0432)^45,54^. Accordingly, the HA has a high purity hexagonal structure. In other words, the XRD pattern of the rGO-HA powders is quite similar to pure HA. According to studies, graphene oxide has a peak in the range of 2theta = 10^48^. After reduction of graphene oxide, this peak is removed and a new one appears around 2theta = 26. This peak is much weaker and wider than the HA (002) peak due to the amorphous structure of rGO. Therefore, the rGO peak is covered by the HA (002) peak which is highly intensified due to its high crystallinity. Table 2 shows the specification of the HA scatter planes obtained. According to the XRD pattern (002), (211), and (300) planes are the main growth planes in HA crystals where, (002) and (300) planes are perpendicular. It is clear that injection of a gas mixture of and thus an increase in total autoclave pressure will increase the crystallinity from 75% (P0) to 86% (P5), 89% (p10) (Eq. 2) and average crystallite size from 32 nm (P0) to 43 nm (P5), 50 nm (P10) (Eq. 3). Increasing the pressure has increased the intensity of the peaks in some directions and decreased in some directions. In the direction of the (002) plane, the injection of gas has reduced the peak intensity (Fig. 2b), but in case (211) and (300) planes the peak intensity has increased (Fig. 2c). Probably due to the increase in pressure applied, the growth of crystals in directions <211> and <300> has continuously increased, and this increase in pressure in direction <002> has first reduced the growth rate and then increased it. For this reason, increasing the intensity of the peaks in the <211> and <300> directions is regular, but in the <002> direction it is irregular. Due to the increase in pressure with gas injection, the growth rate in directions <211> and <300> has increased more than direction <002>, and with the further increase of this pressure, the growth rate has increased in all directions. The peaks are somewhat sharper in the samples that have been synthesized in the presence of injected gas. Also, the peaks have been transmitted slightly to the right, which is probably due to increased pressure from the gas injection. Increasing the crystallinity in the use of injected gas caused the HA to be synthesized with a more precise stoichiometric ratio. As shown in the FESEM image (Fig. 2d,e), the graphene sheets (rGO) are wrinkled and folded while the HA particles are stuck on their surfaces, between them, and on the edges. The HA particles have agglomerated in some places. It is clear that morphology of these particles is nanorod shaped. These nanorods are less than 50 nanometers in diameter but have longitudinal variations. As with similar reports previously published, here is the direction of the growth of nanorods in the C-axis^33,46,49^.

**Table 2:** Specification of the HA scatter planes in rGO-HA powders.

2 Theta (deg)	26	32	32.3	33	39.9	49.6
d-Spacing (nm)	0.343	0.281	0.277	0.271	0.225	0.184
(hkl)	(002)	(211)	(112)	(300)	(310)	(213)

**Figure 3:**
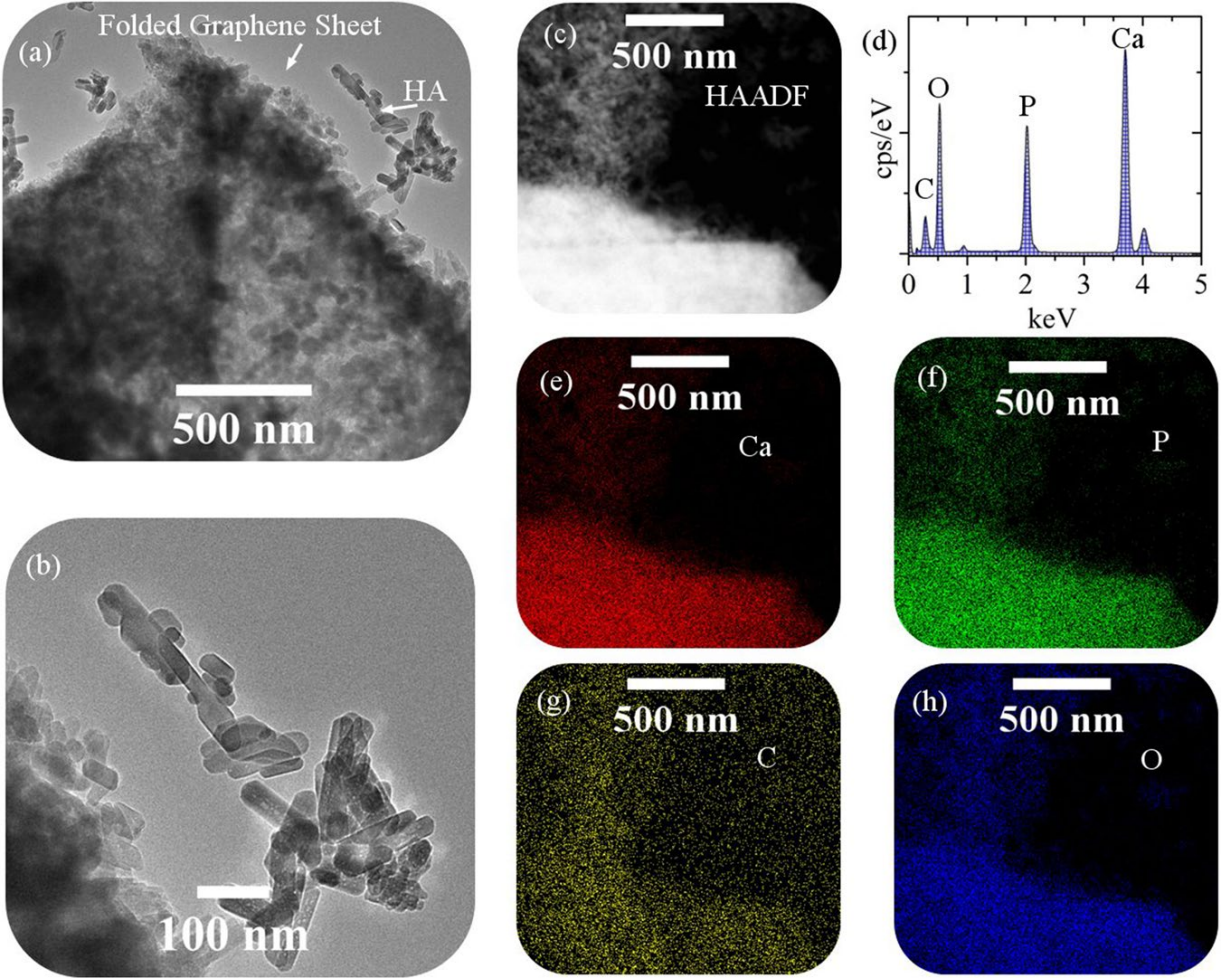
(**a,b**) TEM images, (**c**) HAADF image, (**d**) EDS analysis, elemental map of (**e**) calcium, (**f**) phosphorus, (**g**) carbon, and (**h**) oxygen for the synthesized powders with the presence of injected gas (P10).

Figure 3 shows the TEM images, HAADF image, EDS analysis, elemental map of calcium, phosphorus, carbon, and oxygen for the synthesized powders with the presence of injected gas (P10). Figure 3a shows the high-density of HA particles at the edges of the folded graphene sheets. The size of the HA particles is very fine and their morphology is in the form of rod and prism (Fig. 3b), indicating that there are preferential growth directions in the case of gas injection mode. The TEM images also show that there is a particles size distribution. Smaller particles of HA have remained on the graphene surface due to stronger contact when preparing the samples. Also, these particles have pores due to the polycrystalline structure of HA and the presence of gases. ICP analysis of the residual solution was performed after the hydrothermal process (The findings showed that the amounts of calcium and phosphorus remaining in the solution were very low and negligible). Due to the ratio of calcium to phosphate in the input chemicals was 1.67, the ratio of calcium to phosphate in the synthesized powders is about 1.67. EDS analysis confirms the presence of trace elements (Fig. 3c–h). Elemental maps show that trace elements are homogenously distributed in the powders (P10)^49^.

**Figure 4:**
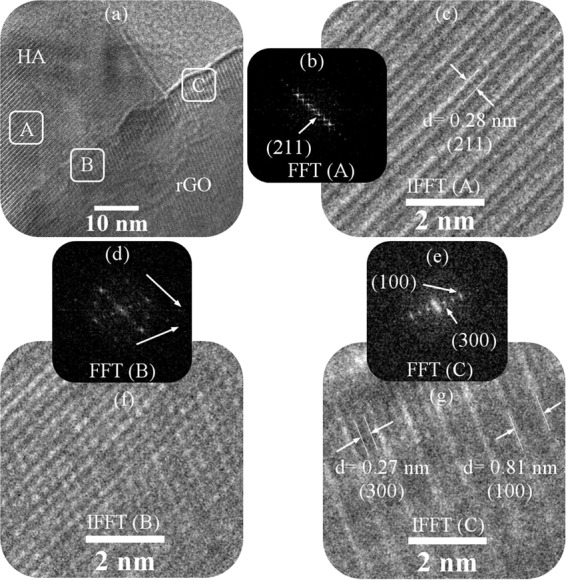
HRTEM image with the FFT and IFFT analysis of composite powders, which is synthesized in the presence of injected gases (P10).

Figure 4 illustrates the HRTEM image with the FFT and IFFT analysis of composite powders, which is synthesized in the presence of injected gases (P10). Three areas were considered in the HRTEM image (Fig. 4a). As the analysis of region A shows (Fig. 4b,c), the HA particles grow along the (211) planes and show a d-spacing of 0.28 nm. But in area C, the (100) and (300) planes are marked with the d-spacing (Fig. 4e,g). The FFT analysis of region B shows the hexagonal structure graphene sheets (Fig. 4d). It is also the place where the crystal planes collide (Fig. 4f)^48,54^.

Schematic 1 shows the crystal structure of HA and a diagram of graphene atomic matching with HA crystalline planes. According to the schematic images (Schematic 1c), the atomic alignment of the crystalline planes with graphene sheets is less than the limit (0.25). Therefore, the interface between the two phases on the graphene surface is likely coherent. According to these findings, (300) planes are likely in contact with the surface of graphene sheets. In this research, during the synthesis of HA, its (300) planes are prior to the (100) planes^48^.

**Scheme 1:**
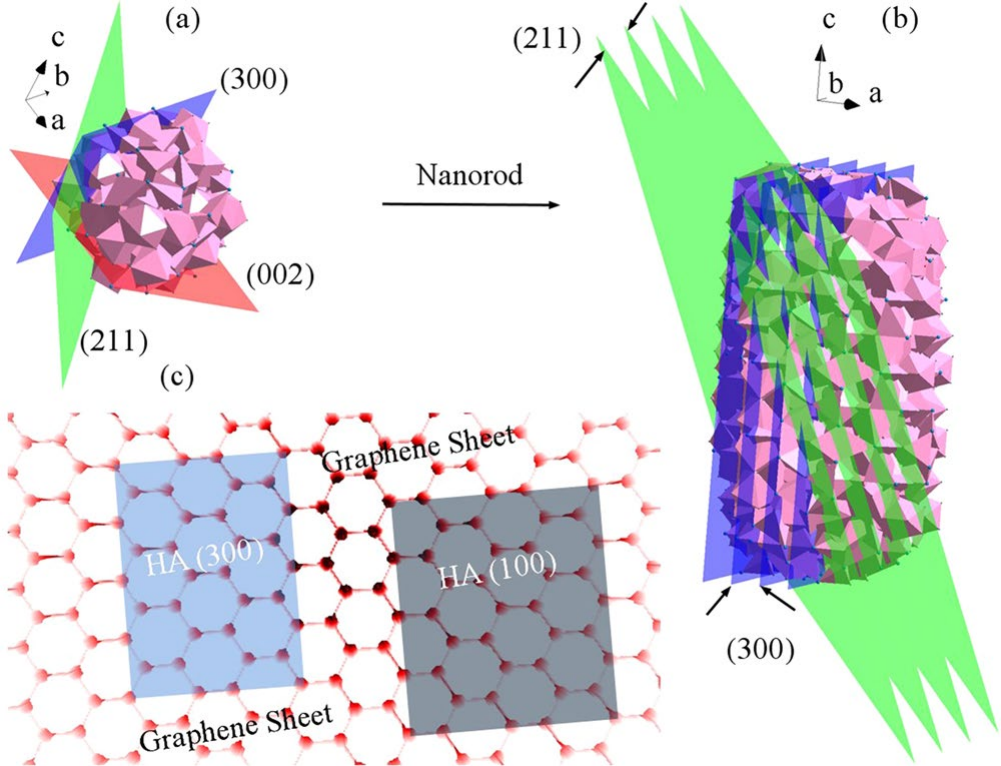
(**a,b**) crystal structure of HA and (**c**) a diagram of graphene atomic matching with HA crystalline planes.

**Figure 5:**
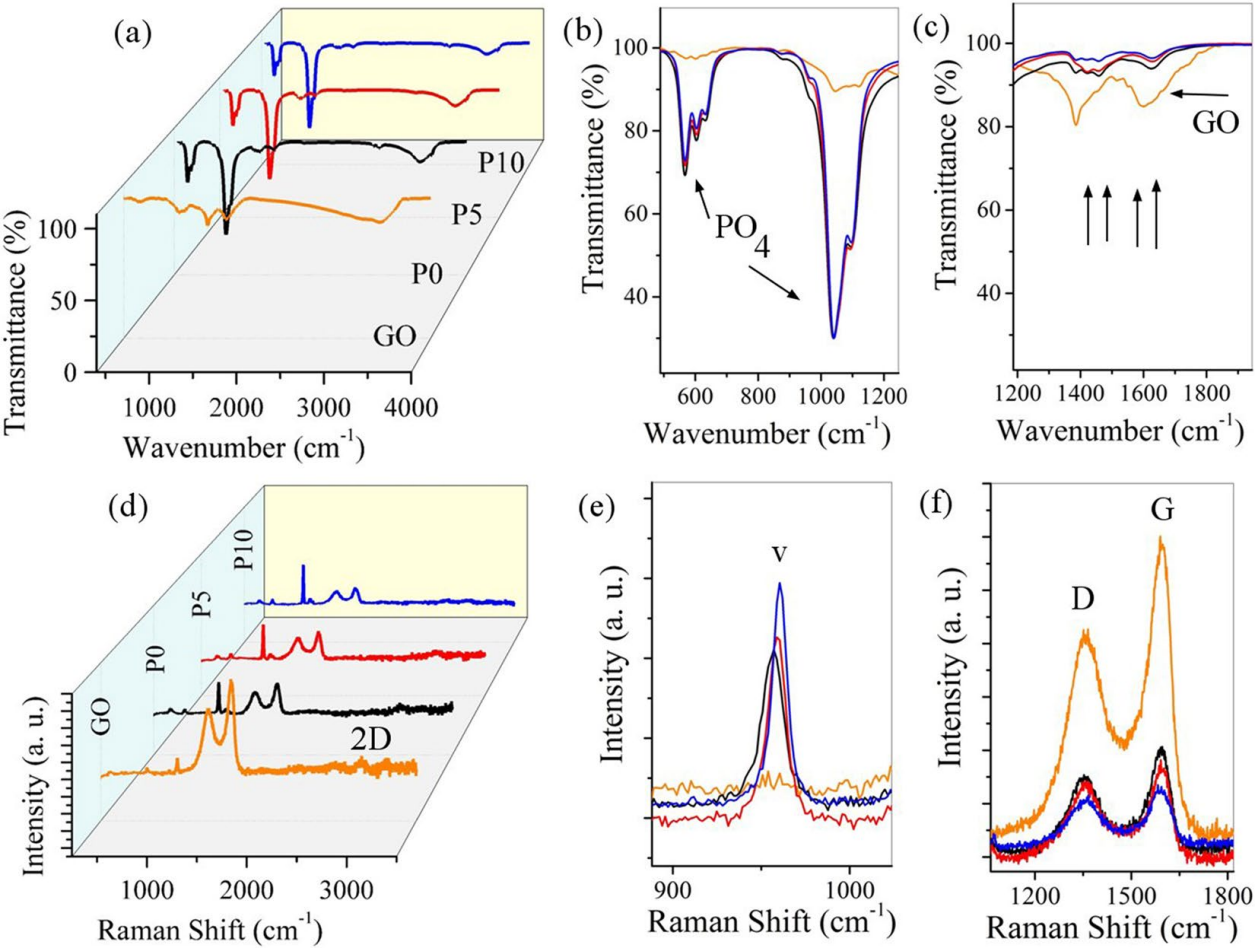
(**a–c**) FTIR analysis and (**d–f**) Raman spectroscopy for synthesized powders and GO.

Figure 5 shows FTIR analysis and Raman spectroscopy for synthesized powders and GO. Table 3 shows the results of FTIR analysis as shown in Fig. 5a–c. The FTIR analysis reveals that the synthesized powders contain graphene sheets and HA. By comparing the GO peaks with the final rGO-HA powders peaks, it is found that the bonds related to the oxygen-containing functional groups on the GO surface have reduced well and some of the peaks have completely disappeared. The peaks at 1395 cm^−1^ and 1730 cm^−1^ are shifted upwards, indicating reduction of GO, since these two peaks are characteristic of GO. As shown in the diagrams, the presence of hydrogen gas in the mixture of gases has led to more reduction. Also, the quality of the peaks obtained for the powders which is synthesized under the gas injection conditions is more accurate and uniform than the other, indicating a better bonds quality^45,49^.

**Table 3:** The results of FTIR analysis for GO and rGO-HA powders.

Wavenumber (cm^−1^)		Bond Mode
565	Related to HA	P-O bending
925, 1035, 1095	Related to HA	P-O(H) stretching vibration
1055	Related to GO Sheets	C-O stretching vibration
1230	Related to GO sheets	C-OH stretching vibration
1395	Related to GO sheets	C-O-H deformation vibration
1620	Related to GO sheets	C=C stretching vibration
1730	Related to GO sheets	C=O stretching vibration
3400- 3500	Related to GO sheets & HA	O-H stretching vibration

The Raman spectroscopy has been done to confirm the presence of rGO. Table 4 shows the results of Raman spectroscopy as shown in Fig. 5d–f. The rGO-related Raman signals in this spectrum are much clearer than the HA signal, although its weight percent in the powders is much lower. The D and G peaks in the rGO have not had any displacement at the Raman spectra, indicating that the rGO-HA powders have been successfully synthesized. Regarding the Raman spectrum of rGO, the peaks intensity ratio (ID/IG) has increased compared to primary GO (ID/IG_(GO)_ ≈ 0.73, ID/IG_(P10)_ ≈ 0.91) which shows the chemical and thermal reduction that has caused structural disorder in the graphene network. It is also clear that the presence of injected gas has led to more reduction of GO^45,49^.

**Table 4:** Raman spectroscopy results for rGO-HA nano-structured powders.

Bond	Raman shift (cm^−1^)	Related materials
v_1_ PO_4_^3-^ (P-O), symmetric stretching peaks	962	Related to HA
D bond, related to the symmetric oscillations of the A1g of carbon atoms with the sp^3^ hybrid	1350	Related to graphene sheets
G bond, related to the shaking of the E2g of carbon atoms phonon with the sp^2^ hybrid	1600	Related to graphene sheets
2D peak, related to the number of layers of the graphene sheets	2700	Related to graphene sheets

Density calculations from the Archimedes method showed that the sample synthesized by the classical method (P0) reached to relative density of 96.66% and the samples that were synthesized in the presence of injected gas (P5, P10) reached to relative density of 95.91% and 96.9%, respectively. The temperature of 950 °C has been chosen to reduce the probability of decomposition of HA crystals and degradation of graphene sheets.

**Figure 6:**
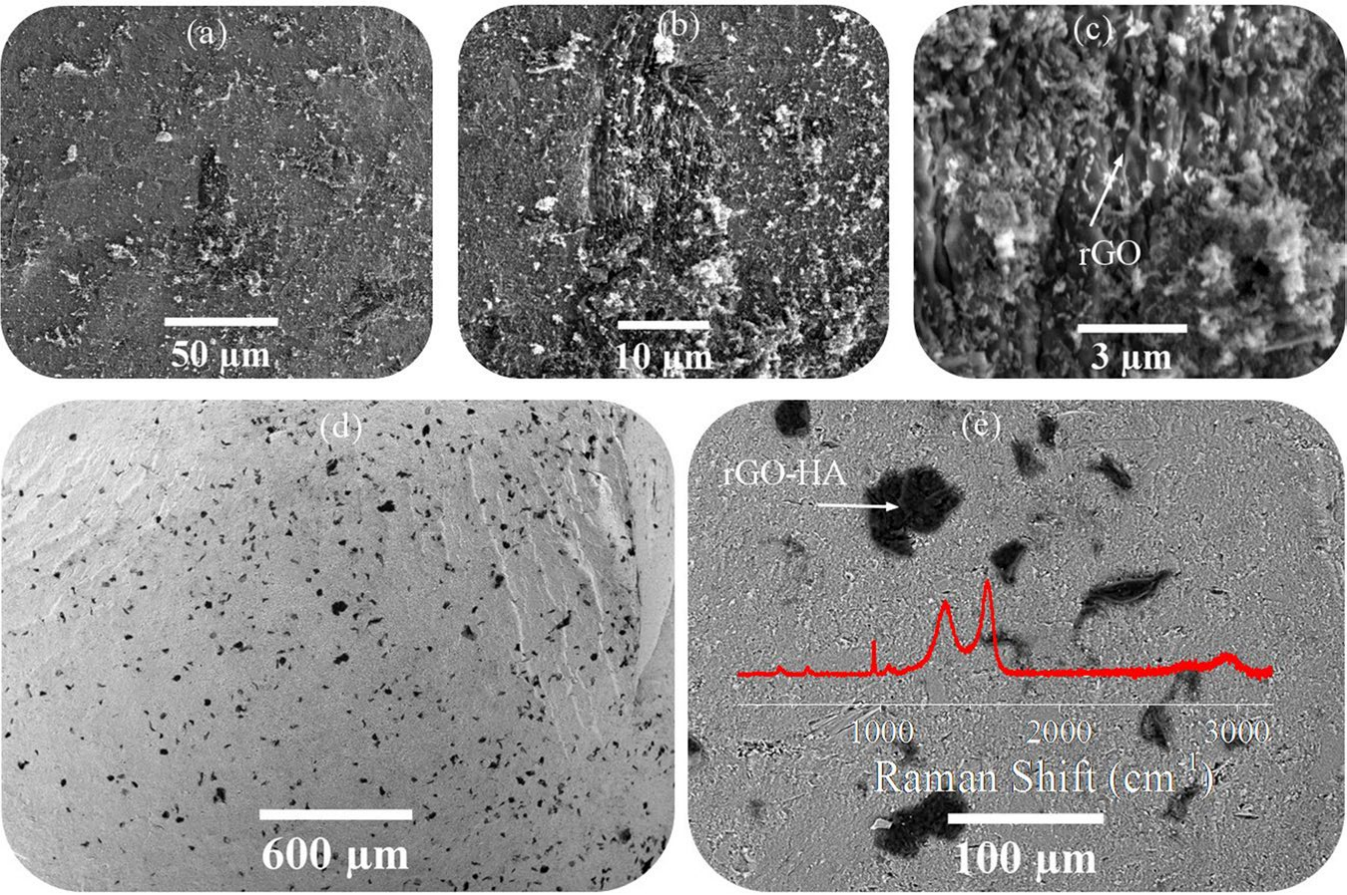
FESEM images of the P10 sample fracture surface after sintering and Raman spectroscopy for P10.

Figure 6 shows the images of the P10 sample fracture surface after sintering and Raman spectroscopy for P10. According to the results of Raman analysis (Fig. 6e), no chemical reaction occurred during sintering and the graphene sheets were rescued from the consolidation process. However, as a result of the sintering process (high pressure and high temperature), the graphene sheets have been partially degraded, reducing the D/G ratio compared to the powder state. Also, some structural change has occurred in the sintered samples, causing the D peak intensity to change. Figure a–c, show the presence of graphene in three dimensions and in Fig. 6d,e, the black spots on the image are the locations of rGO-HA particles, which are distributed as acceptable in the sample volume. The HA nanorods are located on the graphene surfaces and between their layers (Graphene sheets are assembled to create a 3D structure). This porous three-dimensional structure reduces the relative density and mechanical properties of the sintered samples but according to previous studies, the presence of these porosities can increase osteoconductivity for these materials^48^.

**Figure 7:**
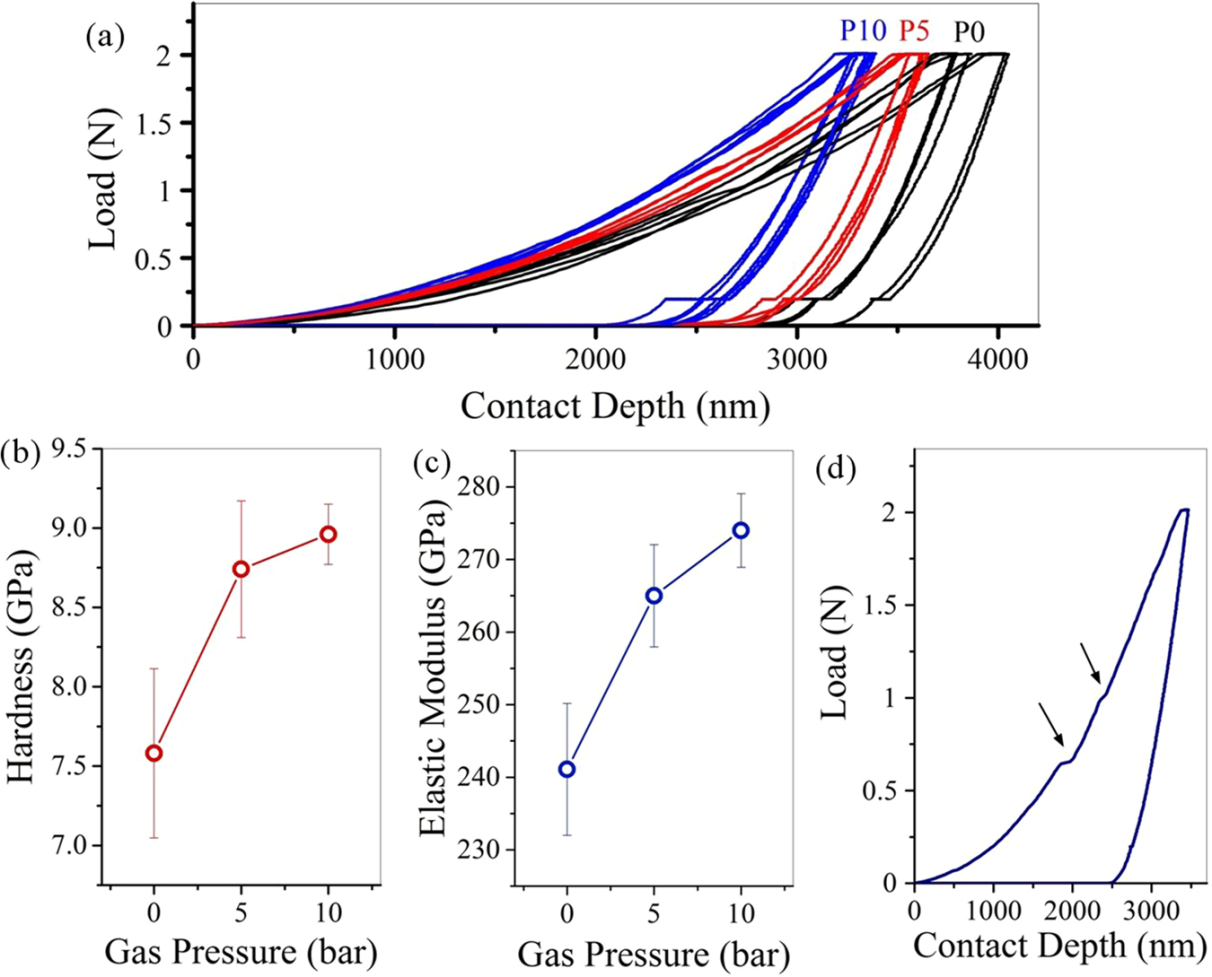
(**a**) force-displacement diagrams of the sintered samples along with (**b,c**) the mechanical properties extracted from these graphs, (**d**) force-displacement curve shows that the Vickers indenter has hit a hole in its path.

Figure 7 shows the force-displacement diagrams of the sintered samples along with the mechanical properties extracted from these graphs. To compare the effect of injected gas during the synthesis of powders on the final properties of the composites, all samples were subjected to a Vickers test. As the curves show the contact depth for P0 is greater than that for P5, P10 samples. In other words, more force is needed to achieve a constant contact depth in P5 and P10. This conclusion is also valid for P10 compared to P5. Considering the same conditions for the preparation of samples, it is likely that another mechanism including a higher degree of GO reduction or higher crystallinity, and a stoichiometric most likely is responsible for this phenomenon. Also, according to these diagrams, the elastic work in P0 is greater than the other samples. Also, the plastics work is slightly higher in P0, but with a smaller ratio, which is obtained from the surface below the curves. In these diagrams, the transition to the left means the improvement of mechanical properties. In Fig. 7d, the force-displacement curve shows that the Vickers indenter has hit a hole in its path. The part shown with the arrow shows the contact depth where the cavity is located. These changes are more evident in samples with more porosity. In some curves, these changes appear several times in a curve. These cases involve some errors in the calculations. The indentation analysis results show that the hardness, the Young’s modulus of P5 and P10 samples are higher than that of P0. Also, P10 showed better mechanical properties than the P5. The reason for this increase in mechanical properties should be examined from two perspectives. First, increasing the hydrothermal pressure increases the crystallinity of the primary powder and improves the properties of the HA, and secondly, the presence of hydrogen gas in the mixture of gases increases the reduction degree of GO and increases the mechanical properties of the graphene sheets. Table 5 shows the hardness and elastic modulus values for the samples^48,54^.

**Table 5:** hardness and elastic modulus values for the samples.

Sample	Gas pressure (bar)	Elastic modulus (GPa)	Hardness (GPa)
P0	0	241.08 ± 9.09	7.58 ± 0.53
P5	5	265 ± 7.05	8.74 ± 0.43
P10	10	274 ± 5.07	8.96 ± 0.19

**Figure 8:**
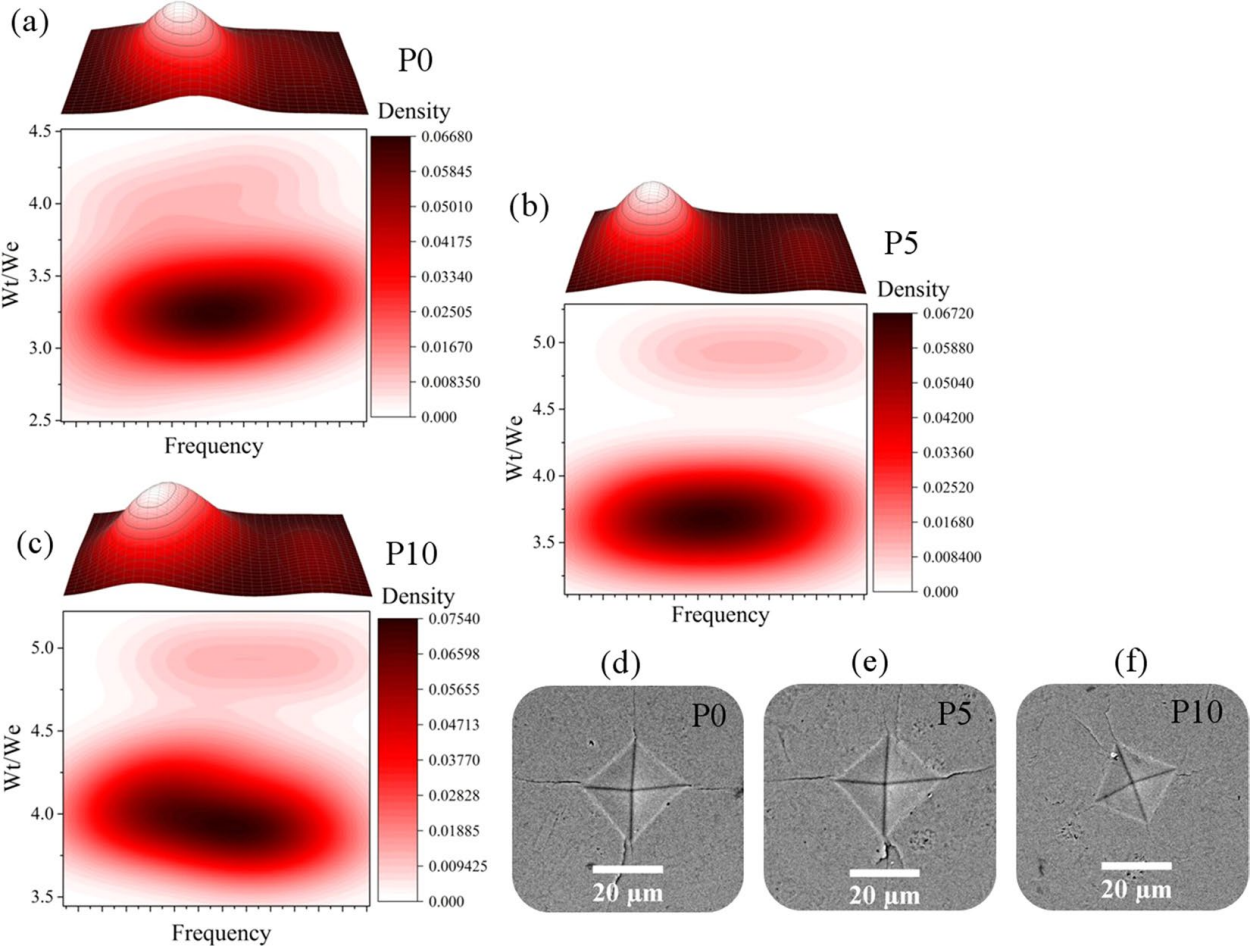
(**a–c**) the three-dimensional and two dimensional diagrams of elastic-plastic work analysis for P0, P5, and P10 nanocomposites after consolidation, (**d–f**) the crack analysis of consolidated samples after Vickers indentation testing.

According to Eq. 1, the fracture toughness (K1C) is directly related to the ratio of total work to elastic work (Wt/We), and is inversely proportional to the crack length. Therefore, comparison of these two factors is useful in analyzing the effect of graphene on fracture toughness. Figure 8 shows the three-dimensional and two dimensional diagrams of elastic-plastic work analysis for P0, P5, and P10 nanocomposites after consolidation along with the crack analysis of consolidated samples after Vickers indentation testing. According to the results, the Wt/We ratio for the P5 and P10 has increased. The size of the cracks in the P5 and P10 nanocomposites are smaller compared to the P0 cracks. In all cases there is a deflection mechanism. This mechanism is due to the presence of nano-sized particles, which increases the strength. In a general conclusion, increasing the Wt/We ratio in the P5, P10 nanocomposites and decreasing the crack length have a dual effect on increasing the P5, P10 nanocomposites K1C compared to P0.

**Figure 9:**
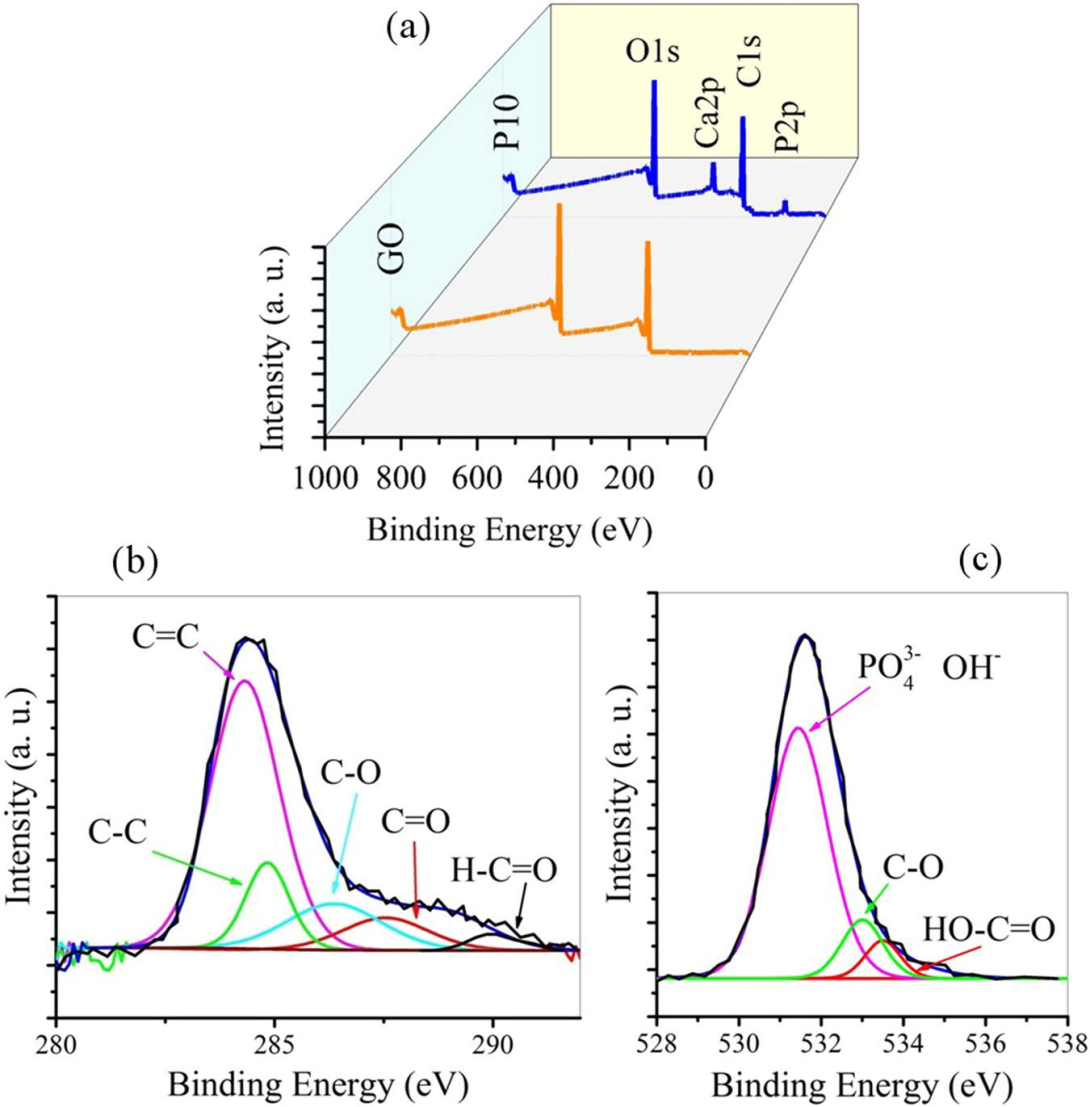
(**a**) XPS analysis of the GO and P10 samples along with (**b**) C 1 s and (**c**) O 1 s high resolution fitted spectra for the P10 sample.

Figure 9 shows the XPS analysis of the GO and P10 sample along with C 1 s and O 1 s high resolution fitted spectra for the P10 sample. XPS analysis is a useful way to evaluate the chemical composition of carbon-based nanomaterials. By comparing Fig. 9a peaks, Ca 2p and P 2p signals confirm the presence of HA phase after sintering process at 950 ^o^C. As a result of these findings, HA has retained its composition after high temperature consolidation and has not been decomposed. According to Fig. 9b,c, the carboxyl groups of GO surface still remain the same as the original GO, due to the strong electrostatic interaction between calcium phosphate and the carboxyl groups present on the surface of GO^48^.

**Figure 10:**
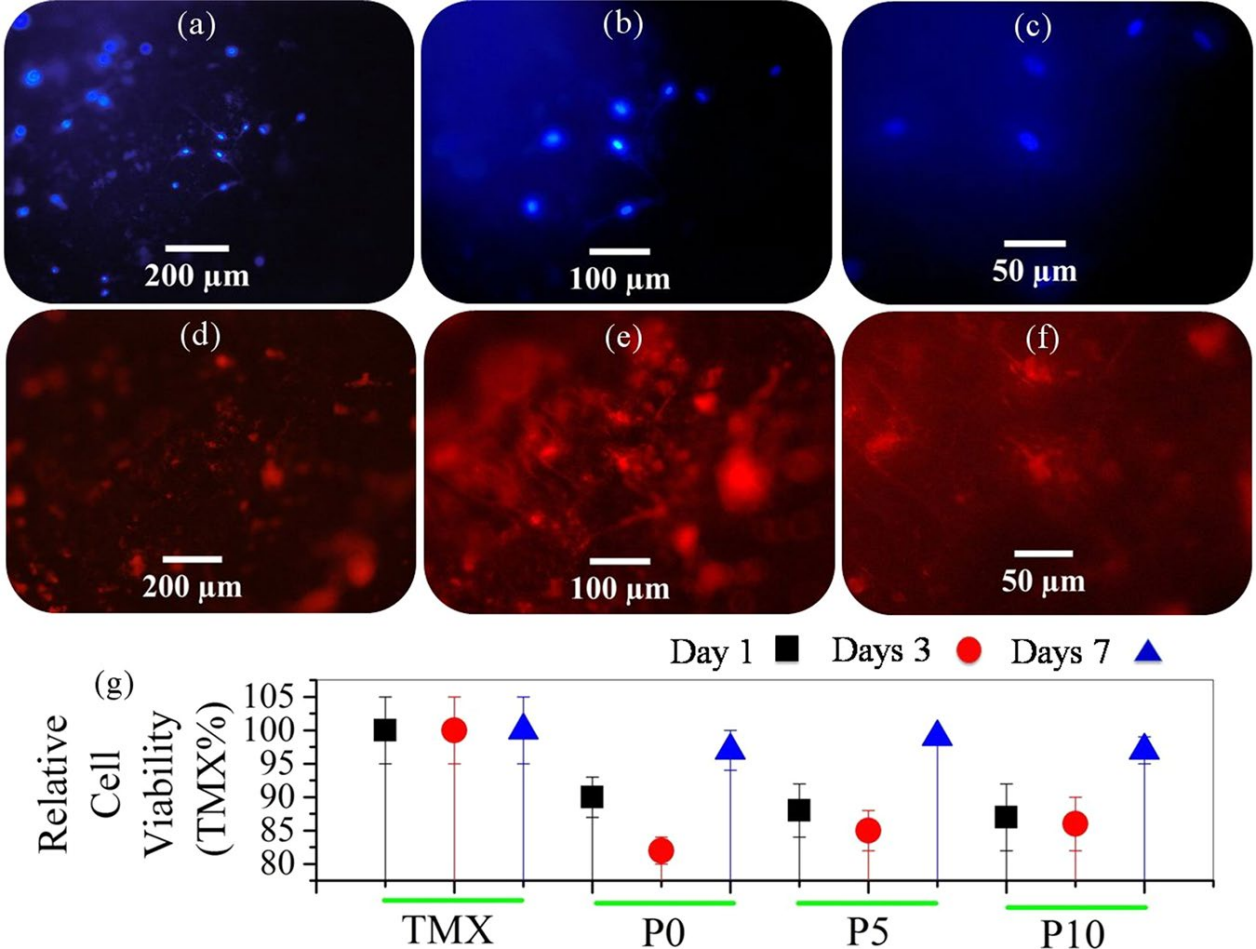
(**a–f**) fluorescent cell culture images on P10 sample after 72 hours and results of the MTT assay.

Figure 10 shows fluorescent cell culture images on P10 sample after 72 hours and results of the MTT assay. The results are similar to those previously published^54^. The only difference is the morphology of the cells that are globular. The presence of rGO sheets, which has become hydrophobic by the loss of its surface agents, may have caused this change. It can be concluded that the leached obtained over a 7-days period showed no significant toxicity to the culture of the osteoblasts.

## Conclusions

The findings showed that the interface between the two phases in the nanocomposite was coherent and the elements were homogenously distributed. The powders synthesized under the injected gas conditions had higher crystallinity and crystallite size. Also, the presence of hydrogen gas further reduced the graphene oxide. These conditions resulted in higher elastic modulus, hardness and fracture toughness of the nanocomposites synthesized by this method. These composites were biocompatible and showed some hydrophobicity compared to pure HA. These findings will be useful for biomedicine applications of these nanocomposites.

## Data Availability

Schematic images of crystals are drawn with Diamond (3.2) software and it can be found through the link: https://www.crystalimpact.com/diamond/. Other schematic images, diagrams, and images were prepared by employing the Powerpoint (2007), Originpro (2016), and ImageJ (1.52 P) softwares that can be found through the links: https://products.office.com/en-us/home, https://www.originlab.com/origin, and https://imagej.nih.gov/ij/download.html, respectively.
